# From hemodynamic stress to inflammatory wall remodeling in intracranial aneurysms

**DOI:** 10.3389/fmolb.2026.1893233

**Published:** 2026-09-14

**Authors:** Junzhe Zhu, Liuxun Hu, Mei Wu, Shiyu Shen, Yingjun Liu, Wei Zhu

**Affiliations:** 1 Department of Neurosurgery, Huashan Hospital, Shanghai Medical College, Fudan University, Shanghai, China; 2 National Center for Neurological Disorders, Shanghai, China; 3 Shanghai Key Laboratory of Brain Function and Restoration and Neural Regeneration, Shanghai, China; 4 Neurosurgical Institute of Fudan University, Shanghai, China; 5 Shanghai Clinical Medical Center of Neurosurgery, Shanghai, China; 6 Department of Pediatrics, Huashan Hospital, Fudan University, Shanghai, China

**Keywords:** endothelial mechanotransduction, endothelial-myeloid interactions, hemodynamic stress, intracranial aneurysms, vessel wall imaging

## Abstract

Intracranial aneurysms are dynamic diseases of the cerebrovascular wall whose growth and rupture cannot be fully explained by size, location, or morphology. Accumulating evidence indicates that aneurysm progression involves coordinated changes in hemodynamic exposure, endothelial phenotype, immune-cell infiltration, vascular smooth muscle cell function, and the extracellular matrix. This review examines how abnormal flow is translated into inflammatory wall remodeling, with particular emphasis on endothelial–myeloid interactions and their downstream effects on mural-cell and matrix homeostasis. Disturbed hemodynamic environments can promote endothelial inflammatory activation and monocyte recruitment, while recruited macrophages adopt heterogeneous functional states that contribute to sustained inflammation, vascular smooth muscle cell dysfunction, and proteolytic injury. Recent single-cell and spatial studies further reveal extensive endothelial, myeloid, and mural-cell heterogeneity within the aneurysm wall. Progressive loss of vascular smooth muscle cell reparative capacity and altered extracellular matrix turnover may ultimately compromise matrix organization and local load-bearing integrity. We also discuss how vessel wall imaging, computational hemodynamics, circulating biomarkers, and omics approaches may help characterize biologically active aneurysms. Together, these findings support a regional view of intracranial aneurysm progression in which mechanical, inflammatory, and structural processes interact within a heterogeneous vessel wall.

## 1 Introduction

Intracranial aneurysms (IAs) are focal dilatations of the cerebral arterial wall that preferentially arise at arterial bifurcations and curved segments of the circle of Willis (Brown and Broderick, 2014; Vlak et al., 2011). Epidemiological studies estimate that unruptured intracranial aneurysms (UIAs) affect approximately 3%–5% of the general population; most remain clinically silent and stable over time (Vlak et al., 2011; Xu et al., 2019). Nevertheless, rupture of IAs results in aneurysmal subarachnoid hemorrhage (aSAH), a catastrophic cerebrovascular event associated with substantial mortality, long-term disability, and socioeconomic burden (Hoh et al., 2023). This clinical paradox—high prevalence but a relatively low rupture rate—poses a major challenge in deciding which aneurysms require preventive intervention, and which can be safely monitored. Current risk assessment still relies largely on clinical factors, aneurysm size, location, and morphology, but these parameters do not fully capture the biological state of the aneurysm wall (Backes et al., 2017; Greving et al., 2014; Samaniego et al., 2019). A central challenge is therefore to identify the biological processes that distinguish stable aneurysms from lesions that undergo progressive remodeling, growth, or rupture.

Traditionally, IAs have been viewed as focal degenerative lesions of the arterial wall that develop in the context of systemic risk factors such as hypertension, smoking, aging, sex-related susceptibility, and genetic predisposition. However, advances in computational fluid dynamics (CFD) (Sadasivan et al., 2013), vascular biology, high-resolution vessel wall imaging (HR-VWI) (Samaniego et al., 2019), and single-cell RNA sequencing (scRNA-seq) (Ji et al., 2024) have broadened our understanding of IA pathobiology beyond conventional morphological assessment. IAs are increasingly recognized as dynamic diseases of the arterial wall in which abnormal hemodynamic exposure, endothelial phenotypic remodeling, immune-cell recruitment, vascular smooth muscle cell (VSMC) dysfunction, and extracellular matrix (ECM) remodeling interact over time (Xu et al., 2019; Meng et al., 2014; Duan et al., 2023; Aoki et al., 2016; Aoki et al., 2017). Importantly, these processes are spatially heterogeneous: distinct hemodynamic environments, inflammatory activity, mural-cell states, and structural wall phenotypes may coexist within the same aneurysm. Understanding how these regional processes interact is therefore important for linking luminal mechanical exposure to wall biology.

Endothelial cells (ECs), which line the luminal interface between circulating blood and the arterial wall, are directly exposed to changes in wall shear stress (WSS), flow direction, and other local hemodynamic cues (Cheng et al., 2025). Under physiological laminar flow, ECs maintain a homeostatic phenotype characterized by anti-inflammatory and antithrombotic signaling, low leukocyte adhesiveness, and stable barrier function. In contrast, disturbed flow patterns at arterial bifurcations and within the aneurysm sac—including oscillatory shear, steep WSS gradients (WSSG), recirculation, and flow stagnation—can alter endothelial mechanotransduction and weaken homeostatic transcriptional programs (Andueza et al., 2020). The role of hemodynamics in IA pathobiology, however, cannot be reduced to a simple high-shear versus low-shear dichotomy (Meng et al., 2014). Localized high WSS and positive WSSG have been associated with early aneurysmal remodeling, whereas relatively low or oscillatory shear environments are frequently observed in established aneurysms with inflammatory or degenerative wall phenotypes (Meng et al., 2014; Metaxa et al., 2010; Wei et al., 2024). These relationships are best interpreted in a regional and phenotype-dependent manner rather than as a validated temporal sequence or as universal WSS thresholds.

Hemodynamic disturbance can also influence inflammatory-cell recruitment through endothelial reprogramming. Activated ECs increase chemokine and adhesion signaling and undergo barrier remodeling, creating conditions that support monocyte adhesion and entry into the aneurysm wall (Aoki et al., 2016; Amersfoort et al., 2022; Aoki et al., 2009). Because normal intracranial arteries generally lack a well-developed vasa vasorum network, the luminal endothelium may represent a particularly important interface between circulating immune cells and the aneurysm wall (Xu et al., 2019). Macrophages subsequently contribute to inflammatory amplification, oxidative injury, proteolysis, and mural-cell dysfunction. Recent IA single-cell studies and broader macrophage literature further challenge a strict M1/M2 interpretation and instead support context-dependent macrophage states shaped by the local vascular microenvironment (Ji et al., 2024; Duan et al., 2023). Endothelial activation and macrophage accumulation may therefore interact to sustain inflammatory wall remodeling, without requiring the assumption of a fully established bidirectional feedback circuit in IAs.

In this review, we examine how abnormal hemodynamic exposure is connected to endothelial inflammatory activation, myeloid-cell recruitment, mural-cell remodeling, and progressive loss of wall integrity in IAs. We also discuss how vessel wall imaging, computational hemodynamics, circulating biomarkers, and omics approaches may be combined to characterize biologically active aneurysms and improve risk assessment.

## 2 Hemodynamic stress and endothelial mechanotransduction

### 2.1 Regional hemodynamic heterogeneity in intracranial aneurysms

IAs preferentially arise at arterial bifurcations and curved segments, where vascular geometry generates marked spatial variation in flow direction, velocity, and near-wall shear (Alfano et al., 2013). Flow impingement at bifurcation apices or outer curvatures may produce localized acceleration, elevated WSS, steep WSSG, and multidirectional shear components, whereas adjacent regions may experience flow separation, recirculation, or delayed washout (Cheng et al., 2025; Cebral et al., 2019; Goubergrits et al., 2012). Once an aneurysmal sac develops, its geometry further redistributes the inflow jet and creates distinct hemodynamic microenvironments across the luminal surface. Consequently, markedly different mechanical conditions may coexist over relatively short distances within the same aneurysm (Cebral et al., 2017).

Several CFD-derived parameters are used to characterize these local environments. WSS represents the tangential traction exerted by flowing blood on the endothelial surface, whereas WSSG describes the spatial variation of WSS along the wall (Reneman et al., 2006; Diagbouga et al., 2018). Oscillatory shear index (OSI) reflects changes in shear direction during the cardiac cycle, while relative residence time (RRT) combines low shear magnitude and oscillatory behavior to identify regions of prolonged near-wall residence (Chen L. et al., 2024; H et al., 2004). Additional descriptors, including low shear area, inflow concentration, WSS divergence, and transverse WSS, capture complementary aspects of flow impingement, stagnation, or multidirectional shear. These parameters describe different features of the local mechanical environment and are therefore most informative when considered together with their spatial distribution and the corresponding wall phenotype.

Evidence from both human observations and experimental models links focal high-shear environments to early aneurysmal remodeling. In a rare longitudinal observation of three patients imaged before aneurysm formation, the arterial regions that subsequently developed aneurysms were exposed to elevated WSS together with positive spatial WSSG (Kulcsár et al., 2011). Experimental studies at the rabbit basilar terminus similarly localized internal elastic lamina loss and early mural remodeling to regions exposed to high WSS and positive WSSG (Metaxa et al., 2010). These findings support a role for concentrated mechanical loading in aneurysm initiation at susceptible arterial sites.

Established aneurysms display a broader range of hemodynamic environments. Regions outside the dominant inflow stream may experience relatively low WSS, oscillatory shear, recirculation, and prolonged residence, and cross-sectional studies have frequently associated these features with inflammatory remodeling or rupture status. In a cohort of 119 IAs, ruptured aneurysms showed lower WSS and higher OSI than unruptured lesions (Xiang et al., 2011). Experimental work further indicates that low-shear disturbed flow can promote endothelial inflammatory activation (Aoki et al., 2016). These observations, however, coexist with evidence that structurally vulnerable regions are not uniformly characterized by low WSS.

Regional flow–wall mapping illustrates this heterogeneity more directly. In 65 surgically treated aneurysms, thin-walled regions tended to align with the principal flow stream and were exposed to higher WSS, whereas atherosclerotic or hyperplastic regions more often colocalized with slow recirculating flow and showed higher oscillatory or residence-time indices (Cebral et al., 2019). Subsequent studies combining intraoperative wall classification with three-dimensional registration similarly reported higher WSS, WSS divergence, and transverse WSS, together with lower RRT, in thin or translucent regions than in thicker wall regions (Veeturi et al., 2022). Thus, high-shear, low-shear, oscillatory, and recirculating environments may coexist within a single aneurysm and correspond to different forms of wall remodeling.

Taken together, current evidence favors a spatially heterogeneous relationship between local hemodynamics and aneurysm-wall phenotype rather than a uniform high-to-low WSS progression. The biological effect of a given flow environment depends on where it occurs and on the cellular and structural state of the wall exposed to it.

### 2.2 Advances and unresolved challenges in patient-specific hemodynamic assessment

Patient-specific CFD has substantially improved the spatial characterization of hemodynamic exposure in IAs, but quantitative estimates remain sensitive to the assumptions used to reconstruct and simulate the vascular geometry. Differences in segmentation, surface smoothing, branch length, inlet and outlet conditions, and rheological assumptions can alter both reconstructed geometry and derived hemodynamic maps (Loly et al., 2025; Paritala et al., 2023; Yang et al., 2023; Fillingham et al., 2024). Surface smoothing, for example, can substantially affect the spatial distribution of time-averaged WSS, while the use of standardized rather than patient-specific blood viscosity may change WSS, WSSG, and OSI without a consistent direction of effect (Paritala et al., 2023; Fillingham et al., 2024). This methodological dependence complicates comparison of absolute values across studies and limits the use of universally applicable “high” or “low” WSS thresholds. Regional and parent-artery-normalized measures can partly reduce interpatient variability, but their interpretation remains dependent on the underlying modeling workflow. Increasing standardization of image reconstruction, mesh and temporal resolution, boundary conditions, and rheological assumptions will therefore be important for comparing hemodynamic findings across cohorts. Complementary approaches such as assimilation of four-dimensional flow MRI (4D-flow MRI) measurements into CFD may further improve patient-specific flow estimation, although current validation remains limited (Ichimura et al., 2025).

A second consideration is the temporal relationship between hemodynamic exposure and aneurysm remodeling. Much of the literature linking low WSS with rupture is based on cross-sectional comparisons between ruptured and unruptured aneurysms, frequently using geometries reconstructed after rupture. Because aneurysm geometry itself strongly influences the flow field, hemodynamic patterns measured after rupture may differ from those present before wall failure. In paired imaging of 21 aneurysms obtained before and after rupture, rupture was accompanied by increases in aneurysm height, volume, aspect ratio, and surface irregularity, together with lower normalized WSS and more stagnant flow (Fujimura et al., 2024). Hemodynamic conditions also evolve during aneurysm growth. In a longitudinal series of 31 growing aneurysms, median WSS decreased after growth, although the magnitude and direction of change varied considerably among lesions (Cornelissen et al., 2022). Low-shear patterns observed in large, irregular, growing, or ruptured aneurysms may therefore reflect, at least in part, the altered geometry produced during disease progression.

Longitudinal imaging provides a more direct opportunity to relate baseline hemodynamic exposure to subsequent remodeling. Recent prospective evidence suggests that these relationships may themselves depend on aneurysm phenotype. In one study, growth of aneurysms smaller than 4 mm was associated with higher time-averaged WSS, whereas growth of larger aneurysms was more closely related to normalized transverse WSS and occurred preferentially at the dome (Fukuda et al., 2025). Such observations indicate that aneurysm size, geometry, and growth pattern can modify the relationship between local flow and remodeling. Longitudinal analyses that calculate hemodynamics from baseline geometry and spatially register subsequent growth onto the original aneurysm surface may therefore provide more informative evidence than comparisons based on post-growth or post-rupture geometries. Nevertheless, growth remains an intermediate phenotype, and its hemodynamic correlates should not be assumed to represent prospective rupture mechanisms.

An additional challenge is that conventional CFD describes mechanical exposure at the luminal boundary, whereas the biological processes associated with instability—including inflammation, matrix remodeling, cellular loss, and wall thinning—occur within the aneurysm wall. Spatially linking these different levels of information has therefore become an important direction in IA research. In a study of 96 unruptured aneurysms, lower WSS measured by 4D-flow MRI was associated with greater aneurysm wall enhancement and permeability-related dynamic contrast-enhanced MRI features (Fu et al., 2025). Another study of 49 unruptured aneurysms found that regions of maximal wall enhancement were frequently located close to CFD-derived low-WSS regions (Jung et al., 2026). These studies demonstrate regional correspondence between luminal hemodynamics and imaging-defined wall changes, although enhancement itself reflects a composite wall phenotype rather than a direct measure of inflammation or mechanical strength.

More detailed flow–wall integration is becoming technically feasible. Multimodal reconstruction has been used to register histological staining, micro-CT, calcification, wall thickness, and CFD-derived WSS within a common three-dimensional aneurysm model, allowing regional mechanical exposure to be compared directly with local wall composition (Niemann et al., 2023). Fluid–structure interaction (FSI) provides a complementary approach by incorporating wall deformation and intramural mechanical loading rather than treating the aneurysm wall as rigid. In a study of 101 sidewall aneurysm geometries, deformable-wall simulations altered near-dome recirculation and several commonly used hemodynamic indices compared with rigid-wall CFD (Goetz et al., 2024). The broader application of such models, however, remains limited by the difficulty of obtaining patient-specific wall thickness, prestress, and spatially heterogeneous material properties *in vivo*.

Hemodynamic parameters are therefore best interpreted as descriptors of the mechanical environment experienced at the luminal surface rather than direct measures of wall degeneration or rupture resistance. Their biological significance depends on when and where they are measured, the geometry in which they arise, and the cellular and structural state of the corresponding wall region. Combining regional hemodynamic mapping with vessel wall imaging, longitudinal morphology, and wall-informed mechanical modeling offers a more direct route for relating luminal mechanical exposure to the biological remodeling discussed in the following sections.

### 2.3 Endothelial mechanosensing and inflammatory reprogramming

ECs form the luminal interface of the aneurysm wall and are therefore the first vascular cells to experience changes in local blood flow. Rather than relying on a single receptor, endothelial mechanosensing is distributed across several interconnected structures, including the glycocalyx, cell–cell junctions, mechanosensitive ion channels, focal adhesions, the cytoskeleton, and nuclear mechanotransduction machinery (Fang et al., 2019; Lim and Harraz, 2024). The endothelial glycocalyx contributes to shear sensing, nitric oxide signaling, permeability control, and leukocyte–endothelial interactions (Reitsma et al., 2007; Askari et al., 2023), while the PECAM-1–VE-cadherin–VEGFR2 junctional complex represents a well-established shear-responsive module. Other receptors, including plexin D1, further illustrate the diversity of endothelial flow-sensing mechanisms described in vascular biology (Tzima et al., 2005; Mehta et al., 2020). Together, these systems allow changes in the luminal mechanical environment to be transmitted into intracellular signaling and transcriptional responses.

Under physiological laminar flow, endothelial mechanotransduction supports a quiescent and vasoprotective phenotype characterized by KLF2/KLF4 activity, NOS3 expression, nitric oxide production, and maintenance of barrier integrity (Sangwung et al., 2017; van Thienen et al., 2006). Disturbed or oscillatory flow can weaken these homeostatic programs and promote inflammatory and remodeling-associated endothelial states (Andueza et al., 2020; Moonen et al., 2022). A single-cell transcriptomic and chromatin-accessibility study in an atherosclerotic vascular model showed that disturbed flow can shift endothelial cells toward pro-inflammatory and mesenchymal-like transcriptional states, indicating that altered mechanical signaling may produce sustained changes in endothelial identity rather than only transient activation (Andueza et al., 2020). Although these cell-state transitions have not been defined to the same extent in IAs, they provide a broader vascular context for interpreting endothelial plasticity in the aneurysm wall.

Mechanosensitive ion channels provide one example of how flow sensing may influence downstream cell behavior. Piezo1 can support KLF2/KLF4-dependent endothelial homeostasis under laminar shear through CaMKII/MEKK3/ERK5 signaling, whereas disturbed-flow studies in other vascular settings have linked Piezo1 signaling to epigenetic and inflammatory remodeling (Zheng et al., 2022; Wu et al., 2024). More directly in IAs, recent evidence showed that high shear stress reduced endothelial Piezo1 expression in human aneurysm tissue and flow-chamber experiments. Reduced Piezo1 activity increased endothelial PDGF-BB expression through YAP/β-catenin signaling and promoted VSMC phenotypic transition through PDGFRβ (Lu et al., 2025). This observation illustrates how altered endothelial mechanosensing may influence not only endothelial function itself but also communication with mural cells.

Inflammatory transcriptional pathways represent a major downstream output of disturbed mechanotransduction. Nuclear factor κB (NF-κB) is particularly well characterized in experimental cerebral aneurysm models. Hemodynamic stress can activate endothelial PGE_2_–EP_2_ signaling, which in turn sustains NF-κB activation and promotes chronic vascular inflammation and aneurysm formation (Aoki et al., 2007a; Aoki et al., 2011). Thus, abnormal mechanical exposure can be converted into a persistent inflammatory endothelial program rather than producing only direct mechanical injury. Flow-responsive transcriptional regulators identified in other vascular systems further suggest that this response is shaped by the balance between protective and inflammatory transcriptional programs (Demos et al., 2022).

One important consequence of this endothelial reprogramming is the production of signals that regulate myeloid-cell recruitment. IA studies have implicated flow-sensitive CCL2 and CCL7 pathways in linking endothelial activation to monocyte/macrophage accumulation and inflammatory activation (Aoki et al., 2016; Wei et al., 2024; Aoki et al., 2009). In parallel, disturbed flow can alter endothelial adhesion and barrier programs, including VCAM-1, ICAM-1, selectins, VE-cadherin, cytoskeletal organization, and permeability-related pathways (Nam et al., 2009; Sluiter et al., 2021). These changes convert the luminal endothelium from a homeostatic barrier into an interface that can support leukocyte adhesion and entry into the aneurysm wall. The regional hemodynamic environments and endothelial mechanotransduction pathways discussed in this section are summarized in Figure 1.

**FIGURE 1:**
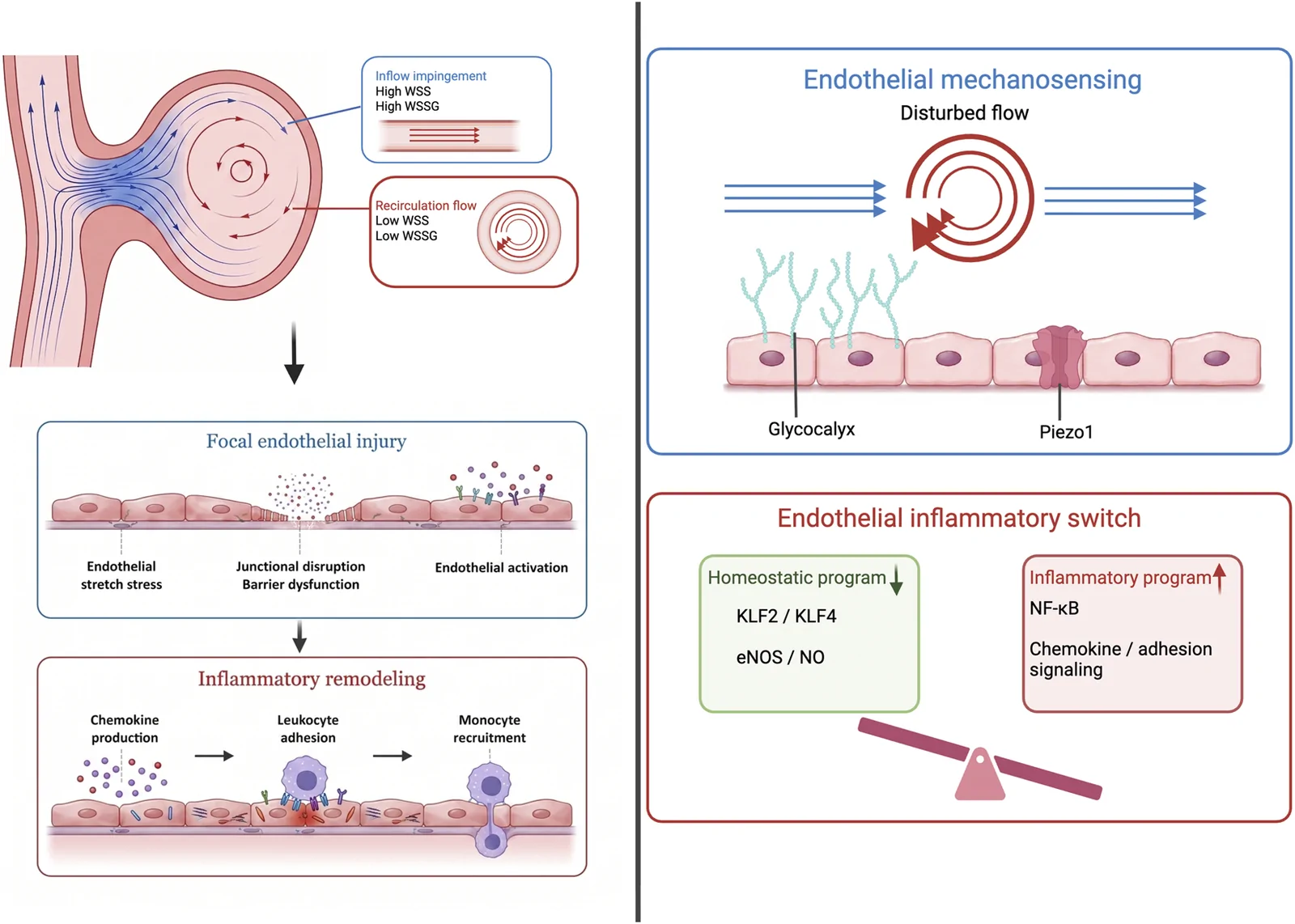
Hemodynamic microdomains and endothelial mechanotransduction in intracranial aneurysms. Abbreviations: WSS, wall shear stress; WSSG, wall shear stress gradient; eNOS, endothelial nitric oxide synthase; NO, nitric oxide; NF-κB, nuclear factor κB.

## 3 Endothelial–myeloid interactions in aneurysm inflammation

### 3.1 Endothelial regulation of monocyte recruitment and transendothelial migration

The luminal endothelium provides a key interface between circulating monocytes and the aneurysm wall. As discussed above, disturbed hemodynamic signaling can shift endothelial cells from a homeostatic barrier phenotype toward a chemokine-producing, adhesive, and more permeable state (Sluiter et al., 2021; Sheinberg et al., 2019). These changes create the conditions required for leukocyte capture and entry into the vessel wall and provide an important link between endothelial activation and myeloid-cell accumulation in IAs.

Leukocyte recruitment across vascular endothelium generally involves a coordinated sequence of tethering and rolling, chemokine-dependent activation, firm adhesion, and transendothelial migration. Selectin-mediated interactions support initial leukocyte capture, while endothelial chemokines activate leukocyte integrins and promote firm adhesion through molecules such as ICAM-1 and VCAM-1 (Ley et al., 2007). Subsequent migration across the endothelial layer requires remodeling of intercellular junctions and the cytoskeleton, involving VE-cadherin, PECAM-1, junctional adhesion molecules, CD99, and tight-junction proteins (Sheinberg et al., 2019; Vestweber, 2015; Shimizu et al., 2019). In IAs, endothelial inflammatory activation, increased adhesion signaling, and macrophage accumulation are consistent with this general recruitment program, although the spatial organization of individual steps within the human aneurysm wall remains less well defined.

Chemokine signaling provides the strongest mechanistic link between activated endothelium and monocyte/macrophage recruitment in IA models. MCP-1/CCL2 promotes monocyte recruitment and macrophage accumulation and contributes to experimental aneurysm formation (Aoki et al., 2009). Flow-sensitive endothelial CCL7 provides an additional pathway: low-shear disturbed-flow conditions increase endothelial CCL7 expression, which promotes macrophage inflammatory activation through CCR1/TAK1/NF-κB signaling (Wei et al., 2024). Together, these findings indicate that endothelial chemokine output may regulate both the entry of circulating myeloid cells and their subsequent inflammatory behavior within the aneurysm wall.

Monocyte heterogeneity may add another layer of selectivity to this process. In vascular immunology, classical CCR2-dependent monocytes are preferentially recruited through CCL2-related pathways, whereas non-classical CX3CR1-associated monocytes display endothelial-patrolling behavior and respond to vascular perturbation through adhesion- and chemokine-dependent interactions (Geissmann et al., 2003; Auffray et al., 2007). Studies in other vascular inflammatory settings further show that CD16^+^ monocytes can interact with activated endothelium through CX3CR1–CX3CL1 signaling and enhance endothelial NF-κB/STAT1 activity together with CX3CL1, ICAM-1, and VCAM-1 expression (Roy-Chowdhury et al., 2021). These observations raise the possibility that different endothelial states may favor recruitment or retention of distinct monocyte populations in IAs, although this has not yet been resolved at the regional or single-cell level.

Endothelial activation therefore shapes myeloid-cell entry through a combination of chemokine signaling, adhesion, and barrier remodeling. Once within the aneurysm wall, recruited monocytes are further shaped by the local inflammatory, metabolic, and structural microenvironment, giving rise to heterogeneous macrophage states.

### 3.2 Macrophage-state heterogeneity and effector functions

Once recruited into the aneurysm wall, monocytes encounter a heterogeneous microenvironment shaped by endothelial-derived chemokines, inflammatory cytokines, oxidative stress, extracellular matrix fragments, signals from mural cells, and, in some lesions, hypoxia (Duan et al., 2023; Lavin et al., 2014; Ono et al., 2023). These local cues can generate macrophage programs with inflammatory, proteolytic, antigen-presenting, metabolic, phagocytic, or reparative features (Lavin et al., 2014; Chen R. et al., 2024). The biological significance of macrophage accumulation therefore depends not only on cell number, but also on functional state, spatial localization, and the surrounding wall phenotype. The cellular origin of these populations is also likely to be heterogeneous, although the relative contributions of recruited monocyte-derived macrophages and resident or perivascular myeloid populations remain incompletely defined in IAs.

Macrophage biology in IAs has traditionally been described using the M1/M2 polarization framework. M1-like macrophages are commonly associated with TNF-α, IL-1β, IL-6, inducible nitric oxide synthase, oxidative stress, and proteolytic activity, whereas M2-like phenotypes are linked to efferocytosis, tissue repair, and inflammatory resolution (Duan et al., 2023; Chen R. et al., 2024). This classification remains useful as a functional shorthand, but it does not adequately capture the diversity of macrophage states within diseased vascular tissue. Experimental IA studies have implicated Axl-dependent M1-like polarization in rupture through STAT1/HIF-1α signaling (Han et al., 2023), whereas human histological analyses have reported an increased M2/M1 ratio in ruptured aneurysm walls (Stratilová et al., 2023). Differences between these observations may reflect variation in disease stage and tissue context, particularly because macrophage phenotypes identified after rupture can be influenced by hemorrhage and acute wall injury.

Single-cell transcriptomic studies provide a broader view of this heterogeneity. Human IA scRNA-seq studies have identified multiple monocyte/macrophage populations within the aneurysm wall, together with inflammatory programs and concurrent endothelial and mural-cell remodeling (Ji et al., 2024; Yu et al., 2025). Studies of atherosclerosis and aortic aneurysm have resolved an even wider range of macrophage states, including inflammatory populations, lipid-handling or TREM2-associated foamy macrophages, and states associated with matrix remodeling or tissue repair (Davis et al., 2022; Dib et al., 2023; Liu Z et al., 2025). These populations should not be assumed to represent identical cell states in IAs, but they illustrate the broader vascular principle that macrophage identity is organized along context-dependent functional programs rather than fixed M1/M2 categories. A state-based view of macrophage heterogeneity in vascular inflammatory diseases is summarized in Figure 2.

**FIGURE 2:**
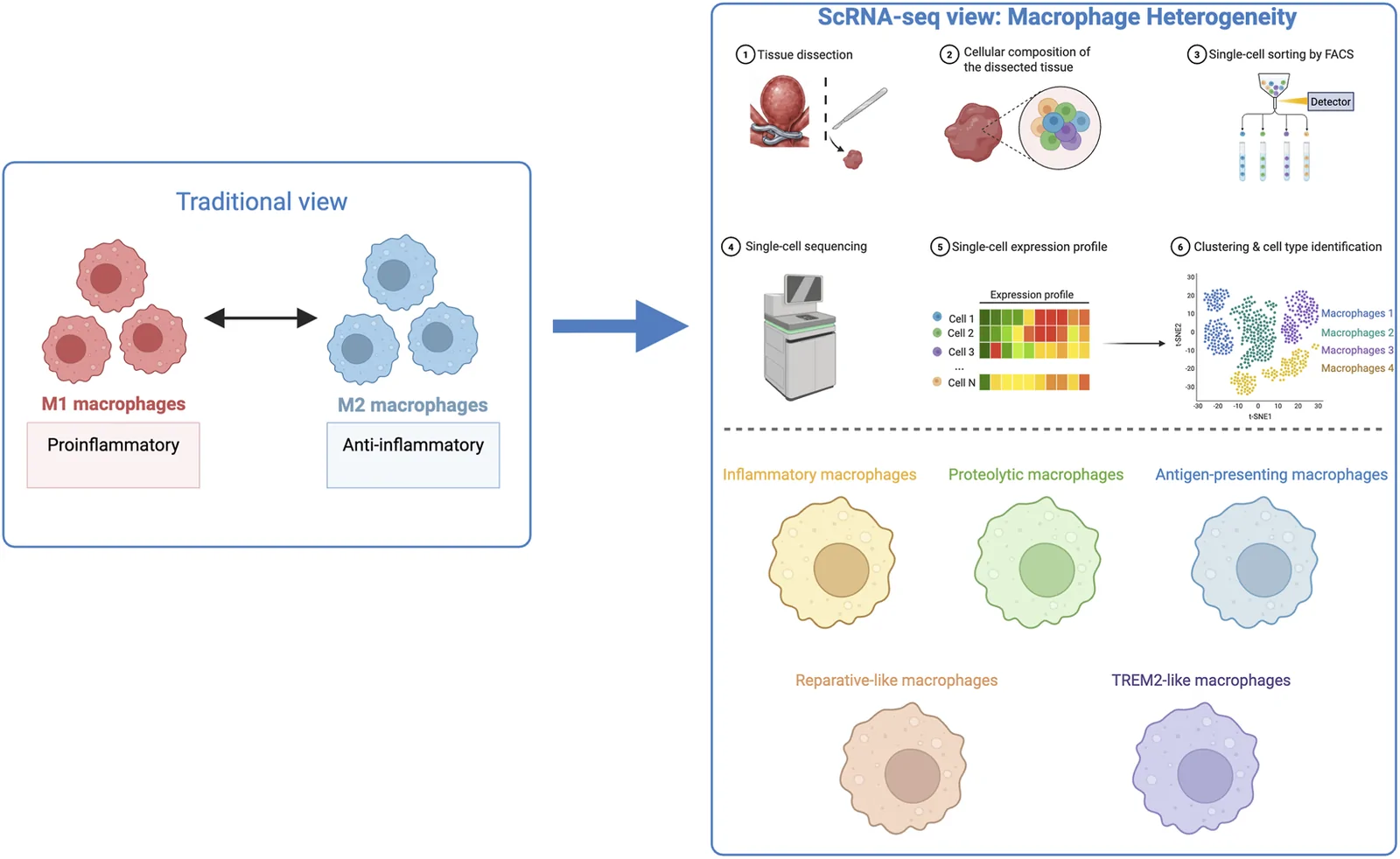
Macrophage-state heterogeneity in vascular inflammatory diseases.

The functional consequences of this heterogeneity are likely to depend on the balance among inflammatory, proteolytic, and reparative programs. Pro-inflammatory cytokines such as TNF-α, IL-1β, and IL-6 can aggravate endothelial dysfunction and VSMC injury, while reactive oxygen species contribute to oxidative damage within the wall (Duan et al., 2023; Ali et al., 2013). Macrophage-derived matrix metalloproteinases (MMPs), particularly MMP-2 and MMP-9, together with cathepsins and other proteases, promote degradation of extracellular matrix components and disruption of collagen–elastin organization. At the same time, phagocytic, lipid-handling, and reparative programs may contribute to debris clearance and tissue repair. In a chronically inflamed aneurysm wall, however, these reparative responses may be insufficient to offset persistent inflammatory and proteolytic injury (Duan et al., 2023; Chen R. et al., 2024).

Macrophage heterogeneity therefore reflects more than variation in inflammatory intensity. Different macrophage states can influence endothelial function, mural-cell homeostasis, and extracellular matrix turnover through distinct but overlapping effector programs. Their spatial distribution within the aneurysm wall may consequently help determine whether local inflammation is accompanied by effective repair or progresses toward persistent tissue injury and structural deterioration.

Representative macrophage-state programs shown in Figure 2 integrate findings from IAs and related vascular inflammatory diseases; not all depicted states have been independently established in human IA tissue.

### 3.3 Endothelial–macrophage interactions and vascular immune crosstalk

Endothelial activation and macrophage accumulation occur in close association during IA inflammation. Endothelial inflammatory signaling can promote myeloid-cell recruitment, whereas infiltrating macrophages contribute to inflammatory amplification and aneurysm progression. Recent experimental evidence further supports this relationship. Central nervous system (CNS)-associated macrophages accumulate within aneurysm walls, and longitudinal molecular imaging has detected increased vascular VCAM-1 and P-selectin expression before hemorrhage; depletion of these macrophages reduced aneurysm formation and rupture (Glavan et al., 2024). These findings support a close functional association between endothelial activation and macrophage accumulation in IAs, although the signals through which macrophages directly modify endothelial phenotype remain less well defined.

Mechanistic studies in other vascular inflammatory diseases provide examples of how reciprocal endothelial–macrophage communication can arise. In atherosclerotic models, disturbed flow promotes endothelial extracellular-vesicle release through MAPK signaling, facilitating monocyte adhesion and transendothelial migration and promoting inflammatory macrophage polarization (Hou et al., 2025). Conversely, macrophage-derived 27-hydroxycholesterol can activate endothelial NF-κB signaling through an ERα/septin 11-dependent pathway, thereby enhancing endothelial activation and monocyte recruitment (Yu et al., 2023). These studies illustrate potential mechanisms through which endothelial and macrophage responses may reinforce one another within inflamed vascular tissue, although the extent to which comparable pathways operate in IAs has yet to be defined.

Recent single-cell studies further suggest that vascular inflammation can be accompanied by persistent changes in endothelial cell state. Human IA single-cell analyses have identified disease-associated endothelial remodeling, including angiogenesis-related programs and ANGPT2-associated changes, but have not defined a discrete immunoregulatory endothelial population (Yu et al., 2025). In atherosclerotic models, disturbed flow combined with hypercholesterolemia induced broader endothelial reprogramming, including an endothelial-to-immune-cell-like transition (EndIT) supported by single-cell profiling and endothelial lineage tracing (Park et al., 2025). Disturbed flow alone produced only partial reprogramming, indicating that mechanical cues may interact with inflammatory or metabolic signals in shaping these endothelial states. In myocardial infarction, single-cell and spatial analyses identified a transient immunoregulatory endothelial cell (IMEC) state characterized by MHC-II-related programs and interactions with T cells (Tombor et al., 2025), while atherosclerosis studies have described IMEC-like endothelial populations with antigen-presentation, costimulatory, and chemokine programs (Cartura et al., 2026). Whether analogous immune-interactive endothelial states emerge in IA walls remains an open question.

Endothelial–macrophage communication also occurs within a broader multicellular wall environment. A recent single-cell and cell-resolution spatial atlas of human brain aneurysms identified loss of structurally supportive smooth muscle cells, emergence of activated perivascular fibroblasts, specialized macrophage infiltration, and prominent fibroblast–myeloid signaling during aneurysm remodeling (Wang J. C et al., 2026). These observations place endothelial and macrophage interactions within a network that also includes mural cells and fibroblasts, whose spatial organization and signaling relationships change as the aneurysm wall remodels. The resulting inflammatory microenvironment may therefore be better understood as a multicellular process in which endothelial, myeloid, and mural-cell states evolve together with progressive loss of wall homeostasis.

## 4 Inflammation-associated loss of wall resilience and structural remodeling

### 4.1 VSMC plasticity and loss of mural reparative capacity

VSMCs are dynamic regulators of aneurysm-wall homeostasis rather than passive structural components of the media. In addition to maintaining vascular tone, VSMCs contribute to ECM synthesis and turnover and exhibit substantial phenotypic plasticity in response to mechanical, inflammatory, metabolic, and matrix-derived cues. The conventional contractile-to-synthetic transition captures only part of this response. In diseased vascular walls, VSMCs can acquire inflammatory, secretory, matrix-remodeling, and injury-associated programs, indicating that phenotypic modulation is better viewed as a continuum of cell states than as a binary transition (Wang et al., 2023; Oka et al., 2020). Early phenotypic adaptation may support tissue repair, whereas persistent reprogramming, impaired matrix maintenance, and progressive cell loss can gradually reduce the reparative capacity of the aneurysm wall.

Mechanical and inflammatory signals can converge on VSMC growth, survival, and phenotypic state. IGF-1 and IGF-1R expression were reduced in human IA tissue, while experimental studies showed that high WSS suppressed VSMC IGF-1 expression, reduced proliferation, and promoted apoptosis. Restoration of IGF-1 signaling enhanced VSMC survival and reduced experimental aneurysm formation through the PI3K/AKT pathway (Li et al., 2026). Other studies have linked VSMC phenotypic changes to inflammatory remodeling. NPY1R-dependent phenotypic transition was accompanied by enhanced inflammatory responses, macrophage accumulation, and M1-like macrophage polarization in experimental IAs (He et al., 2024). These findings indicate that VSMC plasticity is shaped by both mechanical and inflammatory signals and can influence the balance between mural repair and tissue injury.

Phenotypically altered VSMCs may also participate actively in the inflammatory wall microenvironment. Transcriptomic cell–cell communication analyses identified THBS1–CD47 signaling as a potential interaction between secretory VSMCs and macrophages. In coculture experiments, secretory VSMC-derived THBS1 enhanced macrophage inflammatory polarization through CD47/NF-κB signaling (Huang et al., 2026). These observations extend the role of VSMCs beyond matrix-producing structural cells and indicate that injury-associated or secretory VSMC states may themselves contribute to local inflammatory signaling. VSMC remodeling may therefore evolve together with endothelial and myeloid-cell activation within a multicellular inflammatory wall environment rather than occurring solely as a downstream consequence of immune-mediated injury.

Recent human single-cell and spatial studies have further shifted attention from VSMC phenotype alone toward the organization of the mural-cell compartment as a whole. A cell-resolution spatial atlas of human brain aneurysms identified loss of structurally supportive smooth muscle cells together with the emergence of activated perivascular fibroblasts and infiltration of specialized macrophages (Wang J. C et al., 2026). Activated perivascular fibroblasts repopulated diseased wall regions and showed prominent signaling interactions with myeloid cells, linking fibrotic remodeling with local inflammation. These findings extend aneurysm-wall remodeling beyond changes in individual cell states to broader alterations in mural-cell composition and spatial organization. Loss of smooth muscle cells together with expansion of fibroblast-rich regions may alter the balance between matrix production, degradation, and repair, thereby creating regional differences in reparative capacity within the same aneurysm.

Progressive cellular attrition provides a further route through which VSMC remodeling can reduce mural reparative reserve. CircGNAQ is upregulated in human IA tissue and is predominantly localized to VSMCs, where circGNAQ–SRSF1 signaling promotes phenotypic switching and apoptosis; experimental circGNAQ knockdown suppressed these changes and reduced aneurysm formation (Zhou et al., 2026). More recently, HMGB1-dependent signaling has been linked to inflammatory activation, VSMC phenotypic switching, and ferroptosis through the TLR4/NF-κB pathway, while HMGB1 inhibition reduced aneurysm formation and progression in experimental models (Zhu et al., 2026). Persistent remodeling may therefore progressively reduce the population of mural cells capable of maintaining and renewing the load-bearing matrix. VSMC remodeling in IAs may extend from adaptive plasticity to inflammatory or secretory reprogramming, progressive cell loss, and broader reorganization of the mural-cell compartment. As this reparative reserve declines, the aneurysm wall becomes progressively less able to restore ECM organization under persistent inflammatory and mechanical stress, favoring matrix disorganization and structural fragility.

### 4.2 ECM remodeling and loss of load-bearing integrity

Progressive loss of mural repair is accompanied by remodeling of the ECM, which forms the principal structural scaffold of the aneurysm wall. The mechanical contribution of collagen and elastin depends not only on their abundance, but also on their maturation, organization, and continuity within the arterial wall. Confocal Raman microspectroscopy of human IA tissue has demonstrated reduced collagen and elastin signals together with disturbed spatial organization and alterations in protein secondary structure (Gollwitzer et al., 2025). Human collagen-imaging studies have further identified regions enriched in newly synthesized, immature type I collagen (Hackenberg et al., 2026). These findings indicate ongoing matrix turnover in which new collagen deposition may coexist with incomplete matrix maturation and organization. Active matrix synthesis therefore does not necessarily imply restoration of structural integrity when tissue renewal fails to re-establish an effective load-bearing architecture.

Inflammatory proteolysis contributes to this persistent matrix turnover. Macrophage-derived MMP-2, MMP-9, and several cathepsins degrade collagen, elastin, and basement-membrane components during experimental aneurysm progression (Aoki et al., 2007b; Aoki et al., 2008). Neutrophils provide an additional proteolytic source. In a study, neutrophils accumulated around rupture sites and produced MMP-9 with detectable collagenolytic activity (Kushamae et al., 2020). In a separate murine study, neutrophil depletion or inhibition of neutrophil extracellular trap formation reduced aneurysm rupture specifically under estrogen-deficient conditions (Patel et al., 2023). Increased collagen turnover is also detectable systemically in patients: circulating levels of the carboxy-terminal telopeptide of type I collagen (ICTP) were higher in patients with unstable than stable IAs (Hackenberg et al., 2025). Although such circulating markers do not directly localize matrix degradation to a specific aneurysm-wall region, they are consistent with increased collagen turnover in clinically unstable disease. As VSMC-mediated repair declines, persistent degradation of the existing matrix becomes increasingly difficult to offset by new matrix deposition, allowing the wall to remain biologically active while progressively losing structural organization.

ECM remodeling is spatially heterogeneous across the aneurysm sac. Histological studies of human IAs have described marked regional variation in wall thickness, cellularity, inflammation, and matrix organization, with thin hypocellular or degenerative regions occurring alongside thickened, inflammatory, or fibrotic wall phenotypes (Frösen et al., 2004). Local hemodynamic conditions also vary across these regions. In a recent study combining intraoperative wall imaging with patient-specific CFD, thin-walled regions showed higher WSS, time-averaged WSS, WSS divergence, and pressure, together with lower RRT, than adjacent normal-appearing regions (Anbananthan et al., 2025). These observations add a structural dimension to the regional hemodynamic heterogeneity described earlier. Wall thickness alone, however, does not define local structural competence. Collagen maturity and fiber organization, elastin integrity, matrix continuity, mural-cell density, and ongoing repair all contribute to the ability of a given wall region to bear and redistribute mechanical load.

The mechanical environment within the aneurysm wall is distinct from the shear forces acting at its luminal surface. WSS represents tangential traction exerted by flowing blood on the endothelium, whereas the wall itself carries intramural stresses generated by blood pressure, aneurysm geometry, tissue deformation, and local material properties (Morel et al., 2022). The ability of the wall to tolerate and redistribute these stresses depends on wall thickness, ECM architecture, and tissue mechanical properties. Fluid–structure interaction studies provide one approach for linking luminal flow with wall deformation, and deformable-wall simulations can alter local flow patterns and several commonly used hemodynamic indices compared with rigid-wall CFD (Goetz et al., 2024). However, patient-specific estimation of intramural stress remains limited by uncertainty in wall thickness, prestress, and spatially heterogeneous material properties.

Regional wall vulnerability therefore emerges from the interaction between local mechanical loading and the biological state of the wall exposed to it. Regions that retain viable mural cells, mature and organized ECM, and sufficient structural continuity may preserve greater capacity to accommodate cyclic loading. In contrast, mural-cell depletion, persistent proteolysis, immature or disorganized collagen, and disruption of matrix continuity progressively reduce this structural reserve. Similar luminal hemodynamic exposures may therefore have different consequences in different regions of the same aneurysm, depending on the local capacity of the wall to maintain and repair its load-bearing architecture. To distinguish mechanisms directly supported in IAs from those inferred from related vascular diseases or broader vascular biology, the principal lines of evidence are summarized in Table 1.

**TABLE 1:** IA-specific evidence and cross-disease mechanistic context for aneurysm wall remodeling.

Mechanistic process	Evidence directly supported in IAs	Evidence from related vascular diseases or broader vascular biology
Endothelial mechanosensing and inflammatory activation	High shear stress reduces endothelial Piezo1 and promotes YAP/β-catenin–PDGF-BB/PDGFRβ signaling and VSMC phenotypic transition. Hemodynamic stress also activates endothelial PGE_2_–EP_2_–NF-κB signaling and promotes aneurysm formation (Lu et al., 2025; Aoki et al., 2007a; Aoki et al., 2011)	Endothelial mechanosensing involves the glycocalyx, PECAM-1–VE-cadherin–VEGFR2, Plexin D1, and cytoskeletal signaling. Laminar shear supports KLF2/KLF4–NOS3/NO homeostasis, including Piezo1–CaMKII/MEKK3/ERK5–KLF2/KLF4 signaling, whereas disturbed flow promotes inflammatory/mesenchymal-like reprogramming and Piezo1-associated epigenetic remodeling (Andueza et al., 2020; Fang et al., 2019; Lim and Harraz, 2024; Reitsma et al., 2007; Askari et al., 2023; Tzima et al., 2005; Mehta et al., 2020; Sangwung et al., 2017; van Thienen et al., 2006; Moonen et al., 2022; Zheng et al., 2022; Wu et al., 2024)
Monocyte recruitment and transendothelial migration	MCP-1/CCL2 promotes monocyte/macrophage recruitment and aneurysm formation. Low-shear disturbed flow induces endothelial CCL7, which promotes macrophage inflammatory activation through CCR1–TAK1–NF-κB signaling (Aoki et al., 2016; Wei et al., 2024; Aoki et al., 2009)	Leukocyte recruitment involves selectins, ICAM-1/VCAM-1–integrin interactions, and junctional molecules including VE-cadherin, PECAM-1, junctional adhesion molecules, and CD99. Classical CCR2-dependent and non-classical CX3CR1-associated monocytes show distinct recruitment behavior; CX3CR1–CX3CL1 signaling can enhance endothelial NF-κB/STAT1 activity (Sluiter et al., 2021; Ley et al., 2007; Vestweber, 2015; Geissmann et al., 2003; Auffray et al., 2007; Roy-Chowdhury et al., 2021)
Macrophage-state heterogeneity and effector functions	Human IA scRNA-seq identifies multiple monocyte/macrophage populations. Axl–STAT1/HIF-1α signaling promotes M1-like polarization and rupture, whereas human histology shows altered M2/M1 ratios. Macrophage inflammatory, oxidative, and proteolytic programs include TNF-α, IL-1β, IL-6, ROS, MMPs, and cathepsins (Ji et al., 2024; Duan et al., 2023; Han et al., 2023; Stratilová et al., 2023; Yu et al., 2025; Ali et al., 2013)	Atherosclerosis and aortic aneurysm studies identify lipid-handling/TREM2-associated, inflammatory, reparative, and matrix-remodeling macrophage states, supporting a state-based model beyond the M1/M2 dichotomy (Davis et al., 2022; Dib et al., 2023; Liu Z et al., 2025)
Endothelial–macrophage communication and immune-interactive endothelial states	Endothelial activation accompanies macrophage accumulation in IAs; CNS-associated macrophage depletion reduces aneurysm formation and rupture. Human IA single-cell studies identify angiogenesis-related and ANGPT2-associated endothelial remodeling, but not a discrete immunoregulatory endothelial population (Yu et al., 2025; Glavan et al., 2024)	In atherosclerosis, disturbed-flow EC-derived extracellular vesicles promote macrophage inflammatory polarization through MAPK signaling, while macrophage-derived 27-hydroxycholesterol activates endothelial ERα/septin 11–NF-κB signaling. EndIT and IMEC/IMEC-like endothelial states have been described in atherosclerosis and myocardial infarction (Hou et al., 2025; Yu et al., 2023; Park et al., 2025; Tombor et al., 2025; Cartura et al., 2026)
VSMC plasticity and mural-cell remodeling	IA studies implicate IGF-1/IGF-1R–PI3K/AKT, NPY1R-dependent signaling, THBS1–CD47–NF-κB, circGNAQ–SRSF1, and HMGB1–TLR4–NF-κB in VSMC proliferation, phenotypic switching, inflammatory communication, apoptosis, and ferroptosis. Human spatial studies further show loss of supportive SMCs and expansion of activated perivascular fibroblasts (Wang JC et al., 2026; Wang et al., 2023; Oka et al., 2020; Li et al., 2026; He et al., 2024; Huang et al., 2026; Zhou et al., 2026; Zhu et al., 2026)	Broader vascular studies support VSMC plasticity as a continuum extending beyond the contractile-to-synthetic transition, including inflammatory, secretory, and matrix-remodeling states
ECM remodeling and structural deterioration	IA studies show reduced/disorganized collagen and elastin, immature type I collagen deposition, MMP-2/MMP-9- and cathepsin-mediated proteolysis, neutrophil-derived MMP-9, NET-associated rupture, and increased collagen degradation marker ICTP. Human studies also demonstrate regional variation in wall structure and hemodynamics (Cebral et al., 2019; Gollwitzer et al., 2025; Hackenberg et al., 2026; Aoki et al., 2007b; Aoki et al., 2008; Kushamae et al., 2020; Patel et al., 2023; Hackenberg et al., 2025; Frösen et al., 2004; Anbananthan et al., 2025)	No separate cross-disease evidence summarized

Abbreviations: YAP, Yes-associated protein; PDGF-BB, platelet-derived growth factor-BB; PDGFRβ, platelet-derived growth factor receptor β; PGE_2_, prostaglandin E_2_; EP2, prostaglandin E_2_ receptor EP2; PECAM-1, platelet endothelial cell adhesion molecule 1; VEGFR2, vascular endothelial growth factor receptor 2; KLF2/KLF4, Krüppel-like factors 2/4; NOS3, nitric oxide synthase 3; CaMKII, calcium/calmodulin-dependent protein kinase II; MEKK3, mitogen-activated protein kinase kinase 3; ERK5, extracellular signal-regulated kinase 5; MCP-1, monocyte chemoattractant protein 1; CCL2/CCL7, C-C motif chemokine ligands 2/7; CCR1/CCR2, C-C motif chemokine receptors 1/2; ICAM-1, intercellular adhesion molecule 1; VCAM-1, vascular cell adhesion molecule 1; CX3CR1, C-X3-C motif chemokine receptor 1; CX3CL1, C-X3-C motif chemokine ligand 1; STAT1, signal transducer and activator of transcription 1; HIF-1α, hypoxia-inducible factor 1α; TNF-α, tumor necrosis factor α; IL, interleukin; ROS, reactive oxygen species; TREM2, triggering receptor expressed on myeloid cells 2; ANGPT2, angiopoietin 2; MAPK, mitogen-activated protein kinase; ERα, estrogen receptor α; IGF-1, insulin-like growth factor 1; IGF-1R, insulin-like growth factor 1 receptor; PI3K, phosphoinositide 3-kinase; AKT, protein kinase B; NPY1R, neuropeptide Y receptor Y1; THBS1, thrombospondin 1; CD47, cluster of differentiation 47; SRSF1, serine/arginine-rich splicing factor 1; HMGB1, high-mobility group box 1; TLR4, Toll-like receptor 4; SMC, smooth muscle cell; NET, neutrophil extracellular trap.

## 5 Translating wall biology into aneurysm risk assessment

### 5.1 Wall imaging and hemodynamic mapping of wall instability

Current assessment of unruptured IAs relies primarily on clinical history, aneurysm size, location, morphology, and established risk models or decision-support tools, including PHASES, ELAPSS, and UIATS (Backes et al., 2017; Greving et al., 2014; Etminan et al., 2015). These approaches inform treatment and surveillance decisions but do not directly characterize the biological state of the aneurysm wall. As discussed above, wall remodeling is spatially heterogeneous and reflects the local interaction of hemodynamic exposure, endothelial and myeloid inflammation, mural-cell dysfunction, and ECM disorganization. Imaging approaches that capture wall activity and local mechanical exposure may therefore provide information complementary to conventional anatomical risk assessment.

High-resolution vessel wall imaging (HR-VWI) and computational hemodynamic analysis interrogate different but potentially complementary aspects of this process. HR-VWI provides an imaging surrogate of wall activity, whereas CFD characterizes the mechanical environment at the luminal surface (Samaniego et al., 2019). Neither approach should be regarded as a stand-alone measure of rupture risk. Their potential value instead lies in identifying regions where imaging-defined wall abnormalities and distinct hemodynamic environments spatially converge.

Aneurysm wall enhancement (AWE) is the most extensively studied HR-VWI feature associated with aneurysm-wall activity. Systematic reviews and meta-analyses have reported associations between AWE and unstable aneurysm phenotypes (Molenberg et al., 2021). Circumferential enhancement has been reported in evolving aneurysms (Omodaka et al., 2019), while quantitative enhancement metrics may improve the objectivity and reproducibility of AWE assessment (Roa et al., 2021). Longitudinal evidence regarding the prognostic value of AWE has become more informative but remains heterogeneous. An international multicenter cohort of 559 UIAs found that AWE was associated with growth or rupture in crude analysis, but the association was attenuated after adjustment for aneurysm size (van der Kamp et al., 2024). More recently, a prospective multicenter analysis of 1,416 UIAs showed that circumferential AWE remained independently associated with 4-year aneurysm instability after adjustment for size ratio, location, shape, and bifurcation configuration (Liu Q et al., 2025). A subsequent long-term study of 224 UIAs, with a median follow-up of 6.8 years, also found that baseline AWE was associated with subsequent aneurysm instability after adjustment for age and aneurysm size (van der Kamp et al., 2026). These findings strengthen the evidence that AWE, particularly circumferential enhancement, identifies aneurysms with a higher probability of subsequent instability, although its incremental predictive value beyond established clinical and morphological factors still requires further validation. Histopathological correlation studies have associated AWE with inflammatory infiltrates, neovascularization and endothelial markers, atherosclerotic or thrombotic changes, and wall thickness, indicating that enhancement is unlikely to represent a single pathological process (Zhong et al., 2021; Digpal et al., 2024). Short-term longitudinal imaging also suggests that enhancement is relatively stable over time. In a multicenter cohort of 387 UIAs, new onset or resolution of AWE occurred in only a small proportion of aneurysms during approximately 1 year of follow-up (van der Kamp et al., 2025). AWE may therefore reflect sustained wall remodeling in some lesions rather than imminent instability. It is best interpreted as an imaging phenotype of wall activity whose significance depends on enhancement pattern, aneurysm morphology, temporal behavior, and the local biological and mechanical context.

CFD provides a complementary map of luminal mechanical exposure. Parameters such as WSS, WSSG, OSI, RRT, inflow impingement, recirculation, and low-shear area describe different aspects of the local flow environment and should not be treated as interchangeable indicators of aneurysm instability. Studies combining AWE with hemodynamic analysis have reported regional associations between enhancement and particular flow environments, including relatively low-WSS or stagnation-prone regions in some UIAs (Hadad et al., 2021; Zhong et al., 2022). Importantly, these relationships are not uniform across aneurysms or across different regions of the same sac. Recent spatial registration of HR-VWI and CFD in 49 unruptured IAs found that the focus of maximal wall enhancement was located close to a low-WSS region in approximately three-quarters of aneurysms (Jung et al., 2026). Earlier 7-T studies likewise showed that the relationship between enhancement and WSS can vary according to the location of the enhanced region within the aneurysm sac, supporting a regional rather than a universal low-WSS interpretation (Hadad et al., 2021). These observations suggest that the spatial relationship between local flow and wall phenotype may be more informative than either aneurysm-averaged hemodynamic values or global enhancement status alone.

Regional multimodal mapping therefore represents a potentially useful direction for biologically informed risk assessment. Rather than asking only whether an aneurysm is globally enhancing or exposed to a single adverse hemodynamic condition, future studies may examine whether focal or circumferential enhancement, morphological irregularity, thin-wall regions, and specific flow patterns colocalize within the same wall territory. Such approaches remain investigational. HR-VWI acquisition and enhancement quantification, blood-signal suppression, image registration and segmentation, and CFD modeling are not yet fully standardized. The 2026 Society for Magnetic Resonance Angiography consensus specifically emphasized adequate blood suppression in intracranial vessel wall MRI, as residual slow-flow signal can produce flow-related artifacts and apparent wall enhancement (Wang Y et al., 2026). Prospective longitudinal studies combining standardized quantitative wall imaging, robust hemodynamic modeling, and tissue or molecular validation will be required to determine whether regional flow–wall mapping provides predictive information beyond established clinical and morphological factors.

### 5.2 Biomarkers, omics, and multimodal integration

Peripheral biomarkers are attractive because they are accessible, repeatable, and potentially useful for longitudinal monitoring (Wu et al., 2023). A multimodal study integrating HR-VWI, serum cytokine profiling, and histology reported that UIAs with AWE had higher serum IL-1β and TNF-α, lower IL-1 receptor antagonist, and more severe wall remodeling, accompanied by increased CD68 and MMP-2 expression in tissue (Liu et al., 2022). Among routine hematologic markers, the neutrophil-to-lymphocyte ratio (NLR) has been independently associated with circumferential AWE in unruptured saccular aneurysms, including small UIAs (Wu et al., 2022). These findings are of translational interest because they connect accessible circulating markers with vessel-wall imaging, although NLR and circulating cytokines remain systemic indices that can be influenced by infection, comorbid vascular disease, medication, and other non-aneurysm factors.

Omics studies can refine biomarker selection by identifying the cell states that generate these inflammatory or remodeling signals. Human IA single-cell data have shown immune-cell enrichment, myeloid heterogeneity, reduced vascular-cell proportions, and endothelial remodeling (Ji et al., 2024). A study profiled 43,462 cells from IA and control arterial samples and reported increased immune cells, fewer vascular cells, angiogenesis-related endothelial programs, and EC-intrinsic ANGPT2 involvement in vascular dilation in a zebrafish model (Yu et al., 2025). These findings provide a rationale for prioritizing biomarkers linked to specific cellular and remodeling programs—such as disease-associated endothelial states, inflammatory myeloid programs, loss of VSMC reparative capacity, and ECM turnover—rather than relying on nonspecific inflammation alone.

The translational value of these findings will depend on whether molecular signals can be linked to the local wall pathology they are meant to represent. Serum cytokines, hematologic indices, and omics-derived signatures may reflect inflammatory or remodeling activity, but they do not indicate by themselves whether these signals correspond to an enhancing wall segment, an adverse hemodynamic environment, a thrombus-adjacent region, or a structurally thinned area. Spatial validation and imaging–tissue correlation are therefore essential for connecting endothelial, myeloid, VSMC, or ECM-related signatures with anatomically vulnerable wall regions.

## 6 Conclusion and future perspectives

Intracranial aneurysm progression is shaped by the interaction of local hemodynamic forces with a heterogeneous and actively remodeling vessel wall. Endothelial activation links altered flow to myeloid-cell recruitment, while macrophage-state heterogeneity, VSMC remodeling, and disturbed ECM turnover further modify the local wall environment. These processes are unlikely to occur uniformly across an aneurysm, which may help explain why similar luminal geometries or hemodynamic conditions can be associated with different wall phenotypes.

Recent single-cell and spatial studies have added important cellular detail to this picture, but several questions remain unresolved. The organization and functional significance of endothelial, myeloid, fibroblast, and VSMC states within specific wall regions are still incompletely understood, and it remains uncertain whether the immune-interactive endothelial states described in other vascular inflammatory diseases have a direct counterpart in IAs. Resolving these questions will require spatially matched analyses of wall histology, molecular profiles, imaging features, and local hemodynamics.

For clinical translation, the main challenge is to determine whether these biological measurements add information beyond established clinical and morphological risk factors. Longitudinal studies integrating quantitative vessel wall imaging, CFD, circulating biomarkers, and molecular profiling may help identify aneurysms undergoing active wall remodeling and clarify which regional wall phenotypes are most closely associated with subsequent growth or rupture. This may provide a more biologically grounded basis for risk assessment than aneurysm-level morphology or hemodynamic averages alone.
